# New Appliances and Things Medical

**Published:** 1908-06-27

**Authors:** 


### NEW APPLIANCES AND THINGS MEDICAL.

[We shall be glad to receive at our Office, 28 & 29 Southampton Street, Strand, London, W.C., from the manufacturers, specimens of all new preparations and applianceswhich may be brought out from time to time.]

### INVALID AND SURGICAL FURNITURE.

A visit of inspection has been paid to Messrs. Carter's, the invalid and surgical-furniture manufacturers, at their new premises at the corner of New Cavendish Street and Great Portland Street, W. The building is constructed for the special purpose of this business, and contains handsome and commodious showrooms. The operations of this important invalid furniture house have steadily developed in the fifty-five years of its existence, and it can now claim to be the foremost of its kind. It is well known to the medical profession, to hospitals, private institutions, and nurses as an establishment where every description of invalid furniture, and appliances for the alleviation of pain or the promotion of comfort, can be obtained. Many eminent physicians and surgeons are indebted to this firm for their ingenuity in constructing appliances suited to the special needs of their patients.

Messrs. Carter are also specialists in ambulances (motor, horse, and hand). They hold a Royal warrant of special appointment to H.M. the King, another to H.M. the Queen of Sweden, upwards of thirty gold and silver medals for excellence in invalid furniture and ambulance construction, and last April were presented with a Royal warrant of appointment to H.M. the Emperor of Germany. They are the manufacturers of motor-ambulances to the War Office and of the hand-ambulance to the Bischoffscheim Street Accident Service of London.

The varied and extensive show of adjustable chairs, couches, and appliances, from the most costly to the most moderate in price, struck one as being probably the finest and most ingenious to be seen. The following appliances may be mentioned as claiming special attention :—The reading-stand (the "Literary Machine"), which relieves the reader of all strain, complete with desk for making notes, table for inkstand, etc., and attachment for reading-lamp, with a bookcase if desired; a delightful reclining-chair which can be extended as a couch; luxurious easy-chairs and bath-chairs and invalid-carriages; hand-tricycles in a dozen varieties; self-propelling chairs for the use of either or both hands; carrying-chairs; and mechanical bedsteads by which a patient can be turned in any direction or transferred from bed to couch or bath (or *vice versâ.*)

